# Invasion and Dispersion of the Exotic Species *Procambarus clarkii* (Decapoda Cambaridae) in Yeongsan River Basin, South Korea

**DOI:** 10.3390/ani11123489

**Published:** 2021-12-07

**Authors:** Jong-Yun Choi, Seong-Ki Kim, Jeong-Cheol Kim, Jong-Hak Yun

**Affiliations:** National Institute of Ecology, Seo-Cheon Gun 33657, Chungcheongnam-do, Korea; skkim@nie.re.kr (S.-K.K.); jckim@nie.re.kr (J.-C.K.)

**Keywords:** river ecosystem, microhabitat, ecosystem risk, stable isotope analysis, Astacura

## Abstract

**Simple Summary:**

Currently, the dispersion of the exotic species *Procambarus clarkii* in the Yeongsan River basin is a major issue in South Korea, where the majority of domestic crayfish occur in upstream river sections, while *P. clarkii* is mainly abundant in the middle and lower river reaches. In 2018, the species was initially observed only in the Jiseok Stream, a tributary stream of the Yeongsan River. Since then, the species has gradually dispersed in the Yeongsan River basin, and by 2021, the species was recorded in most of the main basin and tributary streams. The larvae of *P. clarkii* were abundant in areas with dense aquatic plants, whereas adults inhabited areas with silt/clay cover. However, *P. clarkii* appears to cause little impact on the freshwater ecosystem, despite its extensive dispersion. The species did not utilize native biological communities as food sources and is unlikely to be consumed by predators.

**Abstract:**

The introduction of exotic species negatively affects the distribution and interactions within local biological communities in an ecosystem and can threaten ecosystem health. This study aimed to provide the basic data required to manage *P. clarkii* in the Yeongsan River basin. We identified the dispersion pattern and evaluated the ecosystem risk of this newly introduced species. The distribution survey investigated *Procambarus clarkii* populations at 25 sites in the Yeongsan River basin over a four-year period. The initial introduction occurred in Jiseok Stream. The larvae of *P. clarkii* were most abundant in areas with a dense aquatic plant cover, whereas adults preferred silt/clay areas. The alterations in the water flow by the river refurbishment project (carried out in 2012) increased their preferred habitats and contributed to *P. clarkii* dispersion. However, stable isotope analysis showed that the dispersion has had little effect on the freshwater ecosystem. The interrelationship between *P. clarkii* (i.e., larvae and adults) and other biological communities has been limited. Although the rapid dispersion by *P. clarkii* in the Yeongsan River basin has not impacted the freshwater ecosystem, further ecological information is required on how to manage *P. clarkii* beyond this early stage of invasion.

## 1. Introduction

Recently, the introduction of exotic species has emerged as an ecological problem arising from continuous exchanges and increased connectivity between countries and regions [1,2]. Although regional environmental factors and climate characteristics clearly limit the distribution of organisms, some species can successfully settle in new ecosystems, utilizing their excellent feeding ability and wide adaptability in various environments [3]. Given that the interactions between biological communities normalize over long time periods, the introduction of exotic species in a region or country is expected to cause significant changes to the existing interrelationship network. The stable settlement of exotic species leads to a reduction and extinction of native species, reducing the regional biodiversity and ecological health [4,5]. It is rare that all exotic species reach a stable settlement, but frequent species introductions and climate change are continuously aiding this process [6]. Previous studies have interpreted this change as the worldwide movement of living things due to global warming [7]. An imbalance in the food network of an ecosystem due to the settlement of exotic species requires a considerable period to recover to a balanced state. Most native species have existed in local ecosystems for a long time, making them quite vulnerable to sudden changes, such as the introduction of exotic species [8]. Empirical studies have suggested that the introduction of higher-order consumers in a food web generates a larger scale of disturbance [9].

Freshwater ecosystems are frequently impacted by the introduction of exotic species, which threatens ecosystem health [10,11]. Ecosystems such as rivers, lakes, and wetlands have limited areas when compared to other ecosystems (i.e., land or marine ecosystems), and a higher frequency of interactions with physical and chemical factors [12,13]. For this reason, freshwater environments are considered ecosystems with complex and heterogeneous microhabitat mosaics. Therefore, freshwater organisms are densely distributed within a limited range, and the biological disturbance caused by the introduction of exotic species may be very damaging. For example, in South Korea, the introduction of exotic fish species such as bluegill sunfish (*Lepomis* *macrochirus*) and largemouth bass (*Micropterus* *salmoides*) clearly led to a decline in native species such as *Pseudorasbora parva* and *Opsariichthys uncirostris* [12]. These two exotic species have caused direct (e.g., predation) or indirect (e.g., reduction in food sources for native species) damage to native species because they invade a wide range of habitats and are excellent at capturing food. In particular, Choi and Kim [14] suggested that the southeastern areas of Korea are dominated by two exotic species (more than 70%). In these areas, fish biodiversity is now very low and cladocerans consumed by the two exotic fish have also changed from pelagic to epiphytic species. This is another example of the imbalances in biodiversity and ecosystem food networks caused by the emergence of powerful predators. Furthermore, red-eared turtles (*Trachemys scripta*), American bull frog (*Rana catesbeiana*), and nutria (*Myocastor coypus*), have all negatively influenced the Korean freshwater ecosystem and are also exotic species that are high-order consumers within the freshwater food web.

Recently, the Yeongsan River area (located in the southwestern section of South Korea) may be undergoing ecosystem disturbance due to the introduction of another exotic species, red swamp crayfish (*Procambarus clarkii*). *Procambarus clarkii* is a freshwater crayfish belonging to Decapoda, Cambaridae, and is native to Louisiana, USA [15]. In China and other countries, it is sometimes consumed by locals, but in Korea, it is rarely consumed. Therefore, we assume that *P. clarkii* was released by a collector or pet owner and it has since dispersed. *Procambarus clarkii* is omnivorous, consuming various food sources, such as aquatic plants and insects, and has adapted to a wide range of freshwater environments. This species is currently widely distributed around the world, including the United States, Asia, and Africa [16]. Reports suggest it has negative effects on native species due to its highly competitive and reproductive characteristics. In Europe, it is included in the list of the top 100 malignant invasive species [17]. In addition, *P. clarkii* mediates various endogenous pathogens, spreading infectious diseases to native species [18]. In Japan, *P. clarkii* is distributed throughout the country, seriously disturbing the freshwater ecosystem, so it is managed as a disturbance species [19]. In South Korea, *P. clarkii* first appeared in Yongsan (district of Seoul) in 1987. In 2006, it was confirmed in Busan and Seoul, including Yongsan [20], but no studies have been conducted since. However, based on the evidence from various countries in which *P. clarkii* is distributed, it is necessary to urgently understand the species distribution and impact on the ecosystem because its invasion in Korea is expected to have a negative impact on the freshwater ecosystems.

In the present study, we investigated the distribution and ecological risk (i.e., influence on native biological communities) of *P. clarkii* in the Yeongsan River basin, South Korea. The aim of this study was to elucidate: (1) the annual distribution of *P. clarkii* in relation to environmental variations, (2) the microhabitat preference of *P. clarkii*, and (3) the trophic position of *P. clarkii* in the native food web. To test these objectives, we surveyed environmental variables, *P. clarkii*, fish, and macroinvertebrates in the Yeongsan River basin. We use our results to discuss the introduction and settlement characteristics of *P. clarkii* in South Korea and suggest management strategies to promote biodiversity in Korean freshwater ecosystems.

## 2. Materials and Methods

### 2.1. Study Description

**Figure 1:**
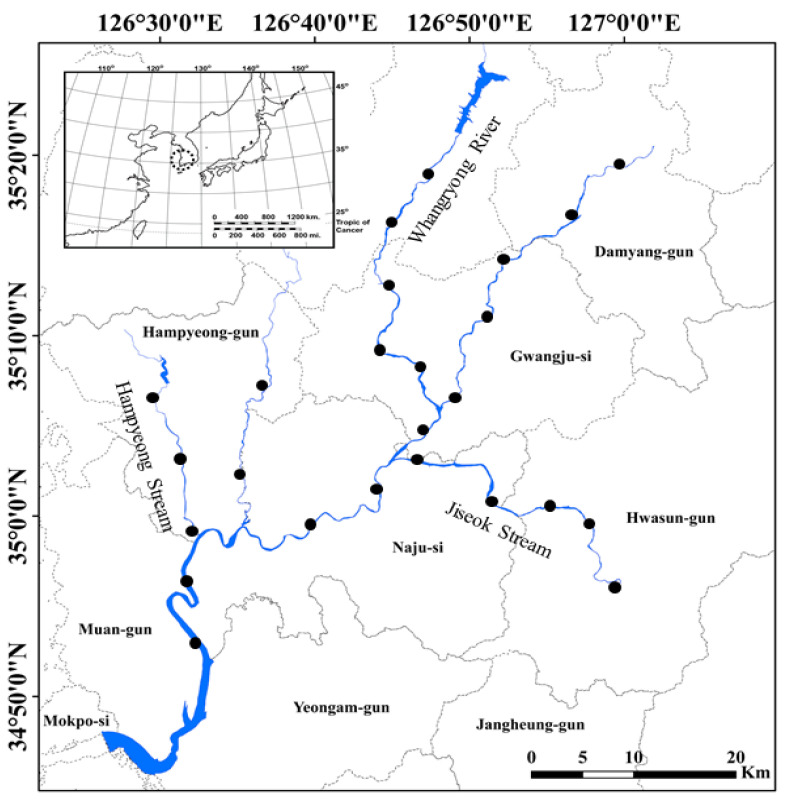
Map of the 25 study sites in Yeongsan River located in southwestern South Korea. The study sites are highlighted by closed circles (●). The small map in the upper left corner is the Korean Peninsula, and the sampling area (Yeongsan River basin) is highlighted with dotted circles.

The Yeongsan River is located in southwestern South Korea, and has a length of 115.5 km and a basin area of 3371 km^2^. The Yeongsan River is the fourth largest river in South Korea (after the Nakdong River, Han River, and Geum River). The major tributary streams such as Gwangju Stream (11.8 km^2^), Hwangnyong River (45 km^2^), Jiseok Stream (34.5 km^2^), Gomakwon Stream (21.4 km^2^), and Hampyeong Stream (15 km^2^) flow into the Yeongsan River basin (Figure 1). These tributaries merge into the main stream of the Yeongsan River to form a larger river basin in the southwestern section of South Korea. In the past, the Yeongsan River basin was affected by flooding, erosion, and salt contamination from agricultural lands near the mid and lower reaches of the river due to tidal influences, but the damage was greatly reduced with the construction of an estuary bank in December 1981.

The Yeongsan River basin is in a temperate climate zone with four distinct seasons (spring, summer, autumn, and winter). Water temperatures range from 10 to 28 °C from spring to autumn. These conditions are suitable for the growth of various aquatic organisms. Unfortunately, the winter average water temperature is 1.6 °C, hindering the growth of most aquatic organisms. However, the number of days exceeding 30 °C in the summer (June to August) has recently increased in this area, changing it to an environment that supports northern-based insects such as *Brachydiplax chalybea flavovittata* [21]. The environmental changes led to the settlement of exotic species, such as *P. clarkii*, in the Yeongsan River basin.

We selected 25 sites located in the Yeongsan River and the main tributary streams (i.e., Hwangnyong River, Jiseok Stream, and Hampyeong Stream) to investigate the distribution of *P. clarkii* (Figure 1). The distance between each site was approximately 5–10 km^2^ using a satellite map (daum kakao map). The upper and lower sections of the main stream and each tributary stream were excluded from sampling as they are not the preferred habitat of *P. clarkii*. At each sampling point, we also collected *P. clarkii* in the littoral zone, which is shallower and has a higher habitat heterogeneity (such as aquatic plants) than the midsection of the river.

### 2.2. Monitoring Strategy

We investigated environmental variables and *P. clarkii* over four years (i.e., from May to June 2018 to 2021) in all 25 study sites located in Yeongsan River, South Korea. Eight environmental variables, including water temperature, percentage saturation of dissolved oxygen (DO), pH, conductivity, turbidity, chlorophyll a (Chl a), total nitrate (TN), and total phosphorus (TP) were measured at each study site. We used a DO meter (model 58; YSI Inc., Yellow Springs, OH, USA) to measure the water temperature and DO. The conductivity and pH were determined using a conductivity meter (model 152; Fisher Scientific, Hampton, NH, USA) and an Orion 250A pH meter (Orion Research Inc., Boston, MA, USA), respectively. To measure the chlorophyll a concentration (Chl.a), 1-L water samples were filtered through 0.45-µm mixed cellulose ester (MCE) membrane filters (Advantech; Model No., A045A047A), and the filtrate was used to determine the concentration of chlorophyll a as described in Wetzel and Likens [22]. We also determined TN and TP spectrophotometrically, based on the method described by Wetzel and Likens [22].

At each site, *P. clarkii* was collected by sampling for approximately 20 to 30 min using a stainless-steel sampler (30 cm width, 600 μm mesh). Using our understanding of the habitat characteristics of the species, we sampled as many *P. clarkii* as possible by sweeping over the sediment surface and over the leaves and stems of aquatic macrophytes. The sampling protocol was consistent in all study sites. The collected *P. clarkii* assemblages and organic materials (including plant debris) were immediately preserved in 10% formaldehyde. In the laboratory, each sample was washed through a 600-μm mesh sieve, and the leaves, stems, and other debris were removed. The resulting material was preserved in 80% ethanol solution.

To understand the spatial distribution of *P. clarkii* larvae and adults under different microhabitat characteristics, we conducted additional collections of *P. clarkii* in two tributary streams (Jiseok Stream and Hampyeong Stream). We identified four different microhabitat types based on the habitat cover, these were: (1) plants, (2) gravel, (3) sand, (4) and silt/clay. At each stream, 25 randomly selected locations of each habitat type (total of 100 sampling points) were surveyed from September to October. For efficient sampling, quadrats (1 m × 1 m) were established at each microhabitats.

### 2.3. Stable Isotope Analysis

Stable isotope analysis was performed to identify the relationship between *P. clarkii* and the abundant biological communities in Jiseok Stream and Hampyeong Stream. Nine separate sampling groups of organisms (planktonic algae, periphyton, cladocerans, dragonfly larvae, damselfly larvae, *L. macrochirus*, *M. salmoides*, *P. clarkii* larvae, and *P. clarkii* adults) were sampled from September to October. The samples were collected three times each month. We collected 5 L of surface water (*n* = 4) with each sample. To process the planktonic algae samples, we initially removed any micro- or macroinvertebrates using a plankton net (32 µm mesh size), and then, the water samples were filtered through GF/F glass fiber filters (0.45 µm; pre-combusted at 500 °C for 2 h). The periphyton-attached macrophyte species (i.e., only the submerged parts of stems, leaves, and roots) and any stone and wood surfaces were gently brushed into a tank filled with distilled water to retain the periphyton samples. Similar to the planktonic algae processing step, plant debris and invertebrates were removed using a plankton net (32 µm mesh size). Cladocerans were separated from the rest of the sample using a total of 10 L of water filtered through a plankton net (70 µm mesh size) with a 10-L column sampler (length: 20 cm; width: 30 cm; height: 70 cm). In addition, invertebrate samples (dragonfly larvae, damselfly larvae, and *P. clarkii*) were collected for approximately 20–30 min using a stainless steel sampler. Two fish species (*L. macrochirus* and *salmoides*) were caught for 30 and 20 min, respectively, using cast nets (7 × 7 mm mesh size) and scoop nets (5 × 5 mm mesh size). For stable isotope analysis, fish muscle tissue samples were collected from the flank near the base of the dorsal fin.

The planktonic algae and periphyton samples were treated with 1 mol L^−1^ HCl to remove inorganic carbon. Then, the samples were rinsed with deionized distilled water to remove the acid. All samples were freeze-dried and ground using a mortar and pestle. All powdered samples were frozen at −70 °C until further analysis. Dried samples were ground into a fine powder using an electronic ball-mill grinder and then stored in clean glass vials.

Carbon and nitrogen isotope ratios were determined using continuous-flow isotope mass spectrometry (CF-IRMS, model-ISOPRIME 100; Micromass Isoprime, GV Instruments Ltd., Manchester, UK). Prior to the analysis, the samples were placed overnight in a sealed CF-IRMS, through which 99.999% He was flowing at a flow rate of a few mL/min. The instrument linearity (dependence of δ^13^C and δ^15^N on signal amplitude at the collectors) was tested daily and confirmed to be <0.03 ‰/nA over the 1–10 nA range. 100  ±  10 μg silver-encapsulated cellulose samples (no carbon was added to samples inside capsules), producing a signal of approximately 4–6 nA at the collectors, were loaded in a 99-position zero-blank CF-IRMS and converted to a mixture of carbon monoxide, carbon dioxide, water, and hydrogen gases over glassy carbon chips in a quartz tube at 1080 °C, within a stream of 99.999% carrier He flowing at 110 mL/min. Data are expressed as the relative per mil (‰) difference between the sample and the conventional standards of Pee Dee Belemnite carbonate (PDB) for carbon and atmospheric N_2_ for nitrogen, according to the following equation:

δ X (‰) = [(R_sample_/R_standard_) − 1] × 1000

where X is ^13^C or ^15^N, and R is the ^13^C:^12^C or ^15^N:^14^N ratio. A secondary standard with a known relationship to the international standard was used as the reference material. The standard deviations of δ^13^C and δ^15^N for 20 replicate analyses of the peptone (δ^13^C = −15.8‰ and δ^15^N = 7.0‰, Merck) standard were ±0.1 and ±0.2 ‰, respectively.

### 2.4. Data Analysis

We performed a stepwise multiple regression analysis to examine the relationship between *P. clarkii* (larvae and adults) with the environmental variables. The influence of *P. clarkii* on the environmental variables was analyzed by initially dividing them into larvae and adults. Furthermore, a one-way ANOVA examined the effects of microhabitat type on the mean density of *P. clarkii*. Tukey’s test provided an additional post-hoc comparison analysis to identify statistically significant differences. All statistical analyses, including analysis of variance (ANOVA) and the stepwise multiple regression, were conducted using SPSS ver. 20 (released 2011; IBM SPSS Statistics for Windows, version 20.0. Armonk, NY, USA: IBM Corp.). Differences and relationships were considered significant if *p* < 0.05.

## 3. Results

### 3.1. Dispersion Pattern of Procambarus clarkii

**Figure 2:**
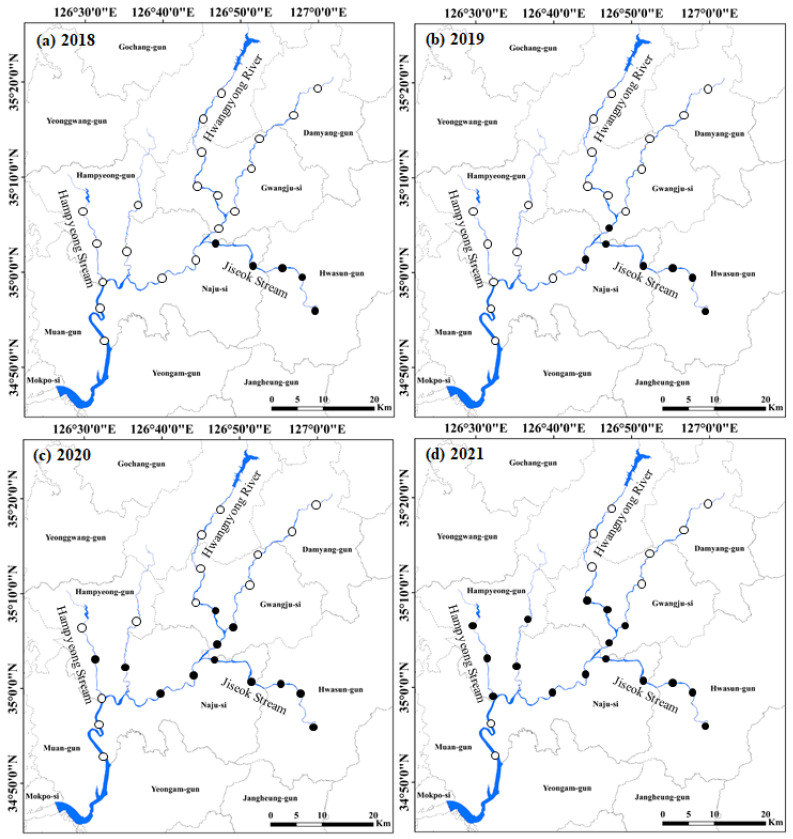
Dispersion pattern of *Procambarus clarkii* during four years (2018~2021) in 25 study sites located at Yeongsan River basin. The study sites where *P. clarkii* was present are indicated as closed circles (●), and the study sites where *P. clarkii* was absent are indicated as open circles (○). (**a**) 2018, (**b**) 2019, (**c**) 2020, and (**d**) 2021.

During the study period (over four years), we observed a clear dispersion pattern by *P. clarkii* in the Yeongsan River basin (Figure 2). In 2018, *P. clarkii* appeared only in Jiseok Stream, a major tributary of the Yeongsan River basin. By 2019, it was sampled at two sites in the main river. In 2020 and 2021, it had dispersed to Hampyeong Stream and Hwangryong River (another tributary of the Yeongsan River). However, *P. clarkii* was not observed at three to four sites located upstream of the main rivers (of both Yeongsan River and Hwangnyong River), or at two points in the downstream sections of the main river.

*Procambarus clarkii* was collected at 19 sites among total of 25 sites, yielding 2 to 16 specimens per site. The highest population of *P. clarkii* was found at four sites in Jiseok Stream (average 13 individuals; ten adults and three larvae), followed by three sites in Hampyeong Stream (with an average of nine; seven adults and two larvae). Since the very first survey in 2018, *P. clarkii* has been continuously abundant in Jiseok Stream. At all other sites, less than five individuals were observed.

The dispersion of *P. clarkii* did not correlate with most of the environmental variables at each site (Table 1). The *P. clarkii* adult distribution correlated with DO and turbidity, and that of larvae highly correlated with turbidity. Furthermore, the environmental variables showed no significant difference between the presence and absence of *P. clarkii* in each year (one-way ANOVA, *p* > 0.05; Table 2).

### 3.2. Distribution of Procambarus clarkii in Different Habitat Types

**Figure 3:**
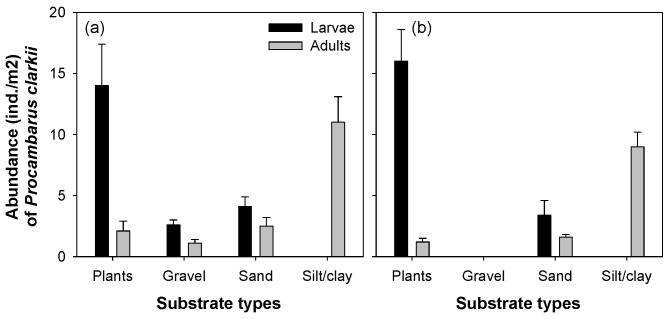
Abundance of *Procambarus clarkii* (larvae and adults) according to four substrate types in Jiseok Stream (**a**) and Hampyeong Stream (**b**); plant, gravel, sand, and silt/clay. (**a**) Jiseok Stream, (**b**) Hampyeong Stream.

The larvae and adult *P. clarkii* had different distribution patterns when compared with the four categories of microhabitats that dominated Jiseok Stream and Hampyeong Stream (Figure 3). The larvae of *P. clarkii* were most abundant in areas covered by aquatic macrophytes (with an average 14 individuals), followed by gravel and sand. Although the larvae of *P. clarkii* did not prefer silt/clay habitats, the adults were most abundant in areas covered by silt/clay (with an average of 12 individuals). Conversely, in the other three microhabitats, the density of *P. clarkii* was relatively low, with an average of only two to three individuals per site. The spatial distribution of *P. clarkii* was similar in both Jiseok Stream and Hampyeong Stream. No *P. clarkii* was found in the gravel substrate in the Hampyeong Stream, while there were some in the Jiseok Stream. In both streams, the density differences of *P. clarkii* residing in each of the four microhabitats were statistically significant (Table 3).

### 3.3. Food Web Structure Incorporating Procambarus clarkii

**Figure 4:**
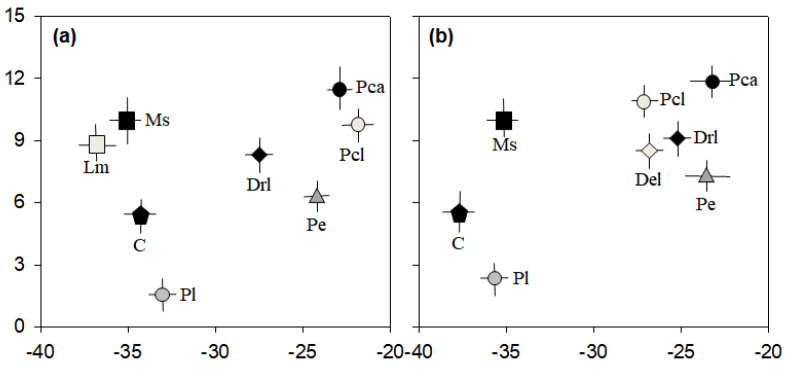
Carbon and nitrogen isotope plots of the samples (*n* = 3) from Jiseok Stream (**a**) and Hampyeong Stream (**b**). Pl, planktonic algae; Pe, periphyton; C, cladocerans; Drl, dragonfly larva; Del, damselfly larva; Lm, *Lepomis macrochirus*; Ms; *Micropterus* *salmoides*; Pca, *P. clarkii* adult; Pcl, *P. clarkii* larva.

The results of the stable isotope analysis revealed that *P. clarkii* had little effect on the abundant biological communities in Jiseok Stream and Hampyeong Stream (Figure 4). The larvae and adults of *P. clarkii* had relatively heavier δ^13^C and δ^15^N values than the other organisms. The local plant communities (such as planktonic algae and periphyton) and animal communities (such as dragonfly larvae, damselfly larvae, and cladocerans) were not consumed by *P. clarkii*. Although the δ^13^C value of *P. clarkii* larvae in Hampyeong Stream averaged −26.4 (which was lighter than in Jiseok Stream) Odonata larvae (dragonfly larvae and damselfly larvae) or periphyton were unlikely to be part of their diet. Conversely, dragonfly and damselfly larvae consume periphyton, and cladocerans rely on planktonic algae. *Lepomis macrochirus* and *M. salmoides* are the dominant fish in Jiseok Stream and Hampyeong Stream, and they mainly consume cladocerans, forming part of the food network route that begins with planktonic algae.

## 4. Discussion

### 4.1. Invasion and Dispersion of Procambarus clarkii

The four year monitoring program of 25 sites located in the main body and tributary of the Yeongsan River basin revealed a distinctive dispersion pattern by *P. clarkii*. Starting in Jiseok Stream in 2018, *P. clarkii* gradually dispersed into major tributary streams such as Hampyeong Stream and Hwangryong River, and the main river (Yeongsan River). We speculate that the environmental changes to this area caused by the River Refurbishment Project in 2012 greatly contributed to the dispersion of *P. clarkii*. The four major rivers (Han, Nakdong, Geum, and Yeongsan Rivers) in South Korea underwent major changes, including the riverside areas, due to the River Refurbishment Project in 2012. The process involved physical changes (such as depth, velocity, and artificialization of the waterfront) and chemical changes (such as dissolved oxygen and pH) in the Yeongsan River basin [23,24]. These artificial environmental changes damaged the previous habitats or induced new creations, causing a definite impact on native biological communities [25]. These changes may induce positive patterns, such as an increase in endangered species [24], or they may lead to negative problems such as the introduction/settlement of exotic species or dominant species. We believe that these habitat alterations played a major role in the introduction and dispersion of *P. clarkii* in the Yeongsan River Basin.

We assume that climate change in this region also contributes to the dispersion of *P. clarkii*. In the last ten years, the air temperatures in the southern sections of the Yeongsan River in South Korea have been gradually increasing, inducing the introduction and settlement of new organisms [21]. For example, the northward movement of southern dragonflies, such as *Sympetrum speciosum* and *Brachydiplax chalybea flavovittata* demonstrate that this area is gradually changing [26]. Choi et al. [21] suggested that the previously observed *Brachydiplax chalybea flavovittata* larvae on Jeju Island were also found in the Yeongsan River area, and the discovery of early stage larvae indicates their settlement in this area. We consider *P. clarkii* to be an exotic species that has utilized environmental changes. Recently, the water temperature in the Yeongsan River basin has rarely dropped below zero (even in winter), creating an environment suitable for *P. clarkii* to survive (above 5 °C) [27].

Considering that *P. clarkii* was first discovered in Jiseok Stream, a major tributary river of the Yeongsan River, we assume that the initial introduction of *P. clarkii* occurred in Jiseok Stream. In Hampyeong Stream (which has similar environmental conditions), it was not found until approximately two years later. In the past, farms had applied for permission to cultivate *P. clarkii* in the area around Jiseok Stream; therefore, it is possible that specimens were introduced into Jiseok Stream for breeding and research on *P. clarkii* before permission was granted. *P. clarkii* is a food source in China and the U.S., but the Korean Ministry of Food and Drug Safety (KMFDS) does not allow *P. clarkii* to be farmed. Therefore, farming is not granted, and there are no *P. clarkii* farms in South Korea. Jiseok Stream has a slow water flow and is covered with littoral vegetation and silt/clay, making it inhabitable for *P. clarkii* [28]. We identified that among the four types of microhabitats that dominate Jiseok Stream, *P. clarkii* larvae prefer areas covered by plants, while adults are abundant in areas covered by silt/clay. The flow rate of Jiseok Stream is artificially controlled by a dam located in the upper reaches, and there is little flow in the remaining seasons (except in summer), making it an ideal habitat for covering plants and silt/clay. This has greatly contributed to the introduction and settlement of *P. clarkii*. In addition, the environmental characteristics in Jiseok Stream are largely consistent with the previous habitat environment in which *P. clarkii* was distributed [29]. However, empirical studies suggest that *P. clarkii* is distributed in relatively diverse environments due to its ability to adapt to a wide variety of biochemical and hydrological habitats [29], with active dispersion capabilities [30,31]. We suggest that *P. clarkii* abundance in any microhabitat (a region covered by plants or silt/clay) in the Yeongsan River basin is due to its habitat preference, which, however, does not hinder its dispersion.

### 4.2. Effect of Procambarus clarkii Invasion on Native Organisms

Our identification of the interrelationship between the major biological communities of the two tributary streams (Jiseok Stream and Hampyeong Stream) by stable isotope analysis revealed that the influence of *P. clarkii* on native organisms was minimal. The carbon isotope concentration consumed by predators was approximately 1‰ and the nitrogen isotope concentration consumed was different (around 3–5‰) from food source of predators [32,33]. *Procambarus clarkii* did not consume the available food sources (e.g., planktonic algae, periphyton, and cladocerans) collected in this study and *clarkii was* not consumed by other predators (*L. macrochirus* and *M. salmoides*). Its carbon and nitrogen isotope concentrations were relatively higher than those of the native organisms, indicating *P. clarkii* was more dependent on other factors than the biological community. In general, epilithic organic matter has a heavier isotope concentration than pelagic organic matter, which has a relatively slow circulation, since the consumption and release by microorganisms continuously occurs [34]. However, based on the isotope value of *P. clarkii*, it is more likely to consume other resources than periphyton. The sediment is composed of organic matter of various origins (e.g., artificial material) as well as animal or vegetable materials, so that it can have a heavier isotope composition that that of pure periphyton [35]. In addition, organic matter produced by artificial sewage or pollutants has a relatively heavier nitrogen isotope concentration than natural organic matter, so that some animal groups consuming this organic matter have heavy nitrogen isotope concentrations [36]. Considering that the δ^15^N values of *P. clarkii* were higher than those of odonata larvae (i.e., dragonfly and damselfly larvae), who mainly consume food sources originating from the bottom layer, it is estimated that *P. clarkii* mainly consumes long-deposited organic matter or animal carcasses. This is a path that clearly differs from the food web path (planktonic algae → cladocerans → fish), which is based on planktonic algae. Although previous studies have suggested that the exploitation of food resources by *P. clarkii* is an important disturbing factor by native organisms (because it can consume a variety of food sources) [37,38], we consider its limited swimming ability to narrow the range of edible food sources.

Therefore, we conclude that the impact of *P. clarkii* in this area is insignificant compared to that of exotic fish species such as *L. macrochirus* and *M. salmoides*, which are already notorious in the Korean aquatic ecosystem. We also expected the dominant fish in the Yeongsan River basin, *L. macrochirus*, and *M. salmoides*, to restrict the population density of *P. clarkii*, but the relationship between the fish species and *P. clarkii* was limited. *L. macrochirus* and *M. salmoides* are exotic fish that were introduced to South Korea in 1970 and ecological disturbance species because they actively consume zooplankton, invertebrates, and native fish [14].

However, they can consume most of the biota living in Korea’s aquatic ecosystem [14], while we did not find that they consumed *P. clarkii*. This may be due to the habitat preferences of *P. clarkii*. *P. clarkii* is distributed mainly in wetlands or littoral areas of streams, impeding predation by *L. macrochirus* and *M. salmoides*. *L. macrochirus* and *M. salmoides* are mainly sight predators [14,15], but *P. clarkii* lives in highly turbid areas and/or habitats with aquatic plants or sediments, making it relatively difficult to see. In addition, the Yeongsan River basin is rich in cladocerans and invertebrates favored by *L. macrochirus* and *M. salmoides*, so there may be no need to prey on *P. clarkii*, which is more difficult to catch. Unfortunately, the low consumption of *P. clarkii* by fish has greatly reduced the likelihood of fish being utilized as a biological control of *P. clarkii*. Most exotic species that have naturalized in various countries (including South Korea) are high-order consumers because they have no predators. *P. clarkii* is also believed to have naturalized in the Yeongsan River Basin without predation.

### 4.3. Control and Management of Procambarus clarkii

Although our results suggest that the dispersion of *P. clarkii* in the Yeongsan River basin has little impact on the native biota, its introduction and settlement may not be positive for this freshwater system. *P. clarkii* has successfully settled on most continents (except Antarctica and Australia) and over time, its abundance was sufficient to change the availability of resources, with its continued exploitation of food resources for native organisms [39,40]. Its settlement in new habitats controls the energy flow in the ecosystem and causes dramatic changes in the organization and function of the ecosystem [38,41]. Various physical or chemical removal measures have been implemented to control *P. clarkii* populations, but there are several cases where management of the permanent populations are difficult [42]. In the 1970s, *P. clarkii* settled in the Southwest Iberian Peninsula [43], where it expanded rapidly [44], impacting agriculture as well as the Iberian native species [45], gastropods [46], native fish communities [47], and amphibians [48,49].

Its active dispersion ability is an issue in the Yeongsan River Basin. Among its extensive habitual adaptations, the high adaptability to water level fluctuations [29] indicates that *P. clarkii* is likely to disperse from the Yeongsan River basin to the surrounding river basins. Unlike other freshwater exotic species that are restricted to disperse in connected waterways, *P. clarkii* can traverse overland [50]. Its overland dispersion is a slow process, and its colonization of isolated areas (i.e., ecosystems with no inflow or outflow, such as wetlands or ponds) is limited to narrow areas around its main habitat, but some studies reported that it can repetitively colonize temporary habitats [51]. Although there is a high possibility that *P. clarkii* will disperse along the main body and the tributary in the Yeongsan River basin, there is a possibility that numerous wetlands around the main river or tributary stream will also contribute to its dispersion to maintain the population. These wetlands have little water flow and high turbidity, limiting the habitat of native crayfish, but supporting *P. clarkii*. Therefore, additional investigations of *P. clarkii* in the wetlands around the Yeongsan River and tributary streams are required. In most rivers and streams in South Korea, several sections contain weirs with sharp falls, which may be difficult for *P. clarkii* to traverse. *P. clarkii* has also a limited swimming ability. Kerby et al. [50] found that natural barriers such as waterfalls may restrict *P. clarkii* dispersion.

## 5. Conclusions

In this study, we identified the habitats that are likely to be occupied by *P. clarkii* and identified management strategies that may limit its dispersion. Currently, *P. clarkii* only exists in the Yeongsan River basin in South Korea. The protection of other habitats from this exotic species may prevent its dispersion to the entire region of Korea, which has occurred in other countries. Although there is a diverse array of information on *P. clarkii* in other countries, further specific research in South Korea, focusing on the ecology of the newly introduced *P. clarkii*, is required. This research should include various hydrological factors that limit their dispersion and ecological information on overland diffusion. These measures are very important in protecting native communities, especially in habitats that have already been colonized or are about to be colonized by *P. clarkii*.

## Figures and Tables

**Table 1:** Summary of the stepwise multiple regression analysis predicting the density of *Procambarus clarkii* (larvae and adults; response variables) with environmental parameters (explanatory variables) in winter. The data were transformed using either arcsine-square root or log (all other variables) transformations prior to the analyses.

*Procambarus clarkii* Types	Explanatory Variables	B_j_	*t*	*p*-Value
Larva	Dissolved oxygen (%)	−0.171	−2.451	0.035
	Turbidity (NTU)	0.139	2.233	0.038
Adult	Turbidity (NTU)	0.227	2.978	0.029

**Table 2:** Environmental variable correlations with the presence or absence of *Procambarus clarkii* in 25 study sites located in Yeongsan River.

Years	Dist. of Pc	WT (°C)	DO (%)	pH	Cond. (µS/cm)	Tur. (NTU)	Chl.a (µg/L)	TN (mg/L)	TP (µg/L)
2018	Presence	21.3 ± 1.6	58.3 ± 12.2	8.1 ± 1.2	235.3 ± 56.3	10.6 ± 3.2	10.6 ± 3.2	1.3 ± 0.2	13.4 ± 2.2
	Absence	21.0 ± 1.2	52.1 ± 10.2	7.9 ± 0.8	258.4 ± 48.4	13.7 ± 4.5	8.4 ± 2.8	1.5 ± 0.3	11.6 ± 2.5
2019	Presence	20.9 ± 1.1	51.3 ± 10.8	7.6 ± 1.3	312.4 ± 48.2	6.4 ± 2.4	11.4 ± 3.1	1.8 ± 0.6	16.3 ± 2.7
	Absence	20.5 ± 0.8	55.4 ± 12.6	7.3 ± 0.7	300.8 ± 40.7	8.1 ± 1.8	9.8 ± 2.4	1.5 ± 0.3	15.2 ± 2.8
2020	Presence	20.3 ± 2.1	46.3 ± 15.3	7.4 ± 0.8	289.3 ± 41.3	12.3 ± 10.4	10.5 ± 4.6	1.1 ± 0.7	11.3 ± 2.0
	Absence	20.0 ± 1.4	51.2 ± 11.9	7.1 ± 1.1	304.5 ± 51.6	11.5 ± 6.3	11.5 ± 3.2	1.0 ± 0.6	13.2 ± 2.1
2021	Presence	21.5 ± 1.5	68.1 ± 16.5	7.6 ± 1.3	284.3 ± 51.2	9.4 ± 3.4	15.4 ± 4.5	1.2 ± 0.6	10.6 ± 1.7
	Absence	21.3 ± 1.6	63.8 ± 12.8	7.5 ± 0.9	289.4 ± 41.8	9.1 ± 2.7	13.7 ± 3.4	1.0 ± 0.8	11.1 ± 1.5

Dist. of Pc, Distribution of Procambarus clarkia; WT, Water temperature; DO, Dissolved oxygen; Cond., Conductivity; Tur., Turbidity; Chl.a, Chlorophyll a; TN, Total nitrogen; TP, Total phosphorus.

**Table 3:** One-way ANOVA results comparing the *Procambarus clarkii* density with the four different habitat substrates (i.e., Plant, Gravel, Sand, and Silt/clay) in Jiseok Stream and Hampyeong Stream.

Streams	*Procambarus clarkii* Types	df	F	*p*-Value
Jiseok	Larva	3	4.462	<0.01
	Adult	3	3.947	<0.01
Hampyeong	Larva	3	4.116	<0.01
	Adult	3	4.015	<0.01

## Data Availability

The data presented in this study are available on request from the corresponding author. The data are not publicly available due to restrictions on the right of privacy.
